# Dynamics and prognostic value of serum neurofilament light chain in Guillain-Barré syndrome

**DOI:** 10.1016/j.ebiom.2024.105072

**Published:** 2024-03-22

**Authors:** Sander J. van Tilburg, Charlotte E. Teunissen, Carolien C.H.M. Maas, Robin C.M. Thomma, Christa Walgaard, Hans Heijst, Ruth Huizinga, Pieter A. van Doorn, Bart C. Jacobs

**Affiliations:** a Department of Immunology, Erasmus MC, University Medical Center, Rotterdam, the Netherlands; b Neurochemistry Laboratory, Department of Clinical Chemistry, Amsterdam Neuroscience, Amsterdam University Medical Centers, Vrije Universiteit Amsterdam, Amsterdam, the Netherlands; c Department of Public Health, Erasmus MC, University Medical Center, Rotterdam, the Netherlands; d Department of Neurology, Erasmus MC, University Medical Center, Rotterdam, the Netherlands

**Keywords:** Biomarker, Guillain-Barré syndrome, Neurofilament light chain, Prognosis, Longitudinal

## Abstract

**Background:**

Neurofilament light chain (NfL) is a biomarker for axonal damage in several neurological disorders. We studied the longitudinal changes in serum NfL in patients with Guillain-Barré syndrome (GBS) in relation to disease severity, electrophysiological subtype, treatment response, and prognosis.

**Methods:**

We included patients with GBS who participated in a double-blind, randomised, placebo-controlled trial that evaluated the effects of a second course of intravenous immunoglobulin (IVIg) on clinical outcomes. Serum NfL levels were measured before initiation of treatment and at one, two, four, and twelve weeks using a Simoa HD-X Analyzer. Serum NfL dynamics were analysed using linear mixed-effects models. Logistic regression was employed to determine the associations of serum NfL with clinical outcome and the prognostic value of serum NfL after correcting for known prognostic markers.

**Findings:**

NfL levels were tested in serum from 281 patients. Serum NfL dynamics were associated with disease severity and electrophysiological subtype. Strong associations were found between high levels of serum NfL at two weeks and inability to walk unaided at four weeks (OR = 1.74, 95% CI = 1.27–2.45), and high serum NfL levels at four weeks and inability to walk unaided at 26 weeks (OR = 2.79, 95% CI = 1.72–4.90). Baseline serum NfL had the most significant prognostic value for ability to walk, independent of known predictors of outcome. The time to regain ability to walk unaided was significantly longer for patients with highest serum NfL levels at baseline (p = 0.0048) and week 2 (p < 0.0001). No differences in serum NfL were observed between patients that received a second IVIg course vs. IVIg and placebo.

**Interpretation:**

Serum NfL levels are associated with disease severity, axonal involvement, and poor outcome in GBS. Serum NfL potentially represents a biomarker to monitor neuronal damage in GBS and an intermediate endpoint to evaluate the effects of treatment.

**Funding:**

10.13039/501100004243Prinses Beatrix Spierfonds W.OR19-24.

**Research in context**

**Evidence before this study**

Neurofilament light chain (NfL) is an established biomarker for neuronal damage, and increasing evidence supports its diagnostic and prognostic value, and/or its role in monitoring treatment response in several neurological disorders. Previous studies have reported increased NfL levels in patients with GBS compared with healthy controls, and high serum NfL levels were associated with disease severity and subtype in GBS. However, no longitudinal studies had been performed in the acute phase of GBS. Consequently, the dynamics and prognostic value of NfL during the acute phase of GBS are unknown.

**Added value of this study**

A longitudinal study was conducted on a well-defined cohort of patients with GBS previously included in a randomised controlled trial (SID-GBS) that evaluated the effect of a second course of intravenous immunoglobulin (IVIg). Serum NfL levels were highly variable; however, most patients had elevated serum NfL at admission that further increased in the next 2–4 weeks, followed by a slow decline, in parallel with the monophasic clinical course of GBS. These results indicate that axonal involvement may occur to some extent in most, if not all, patients with GBS. Interestingly, at the initial stage of the disease (before treatment), there was no direct correlation between serum NfL and disease severity or subtype. However, we found serum NfL levels before treatment had prognostic value for the ability to walk unaided, independently of other established clinical factors for GBS. Between 1 and 4 weeks after initiation of the first course of IVIg, clear associations were observed between serum NfL and disease severity and electrophysiological subtype. Notably, no significant differences in serum NfL were observed among the two treatment arms of the SID trial, in alignment with the overall lack of significant clinical benefit in the trial.

**Implications of all the available evidence**

Our findings demonstrate that the dynamics of serum NfL may represent an easily accessible biomarker for monitoring the disease course and classifying the subtype of GBS, and may also have value as an intermediate endpoint in treatment trials.

## Introduction

Guillain-Barré syndrome (GBS) is an acute immune-mediated polyradiculoneuropathy, with an estimated incidence of 1–2 per 100,000 person-years.^1^ The typical course of GBS is monophasic, characterised by rapid progression of bilateral limb weakness that can last up to four weeks, followed by slower, and often incomplete, clinical recovery over several months or longer.^2^ Effective treatments for GBS include intravenous immunoglobulin (IVIg) and plasma exchange.^3^ However, the response to treatment is highly variable among patients and many individuals continue to experience long-term residual deficits and complaints. Prognostic models, such as the modified Erasmus GBS Outcome Score (mEGOS), are based on demographic and clinical predictors.^4^^,^^5^ Incorporation of additional biomarkers may further enhance the accuracy of these models for predicting clinical outcome and treatment response.

Neurofilament light chain (NfL), a scaffolding protein subunit of the neuronal cytoskeleton, is a promising biomarker for axonal damage in both central and peripheral nervous system disorders.^6^, ^7^, ^8^, ^9^ Following axonal degeneration, NfL is released into the extracellular fluids, including CSF and peripheral blood.^7^ Previously, both NfL and neurofilament heavy chain (NfH) were shown to be elevated in the CSF of patients with GBS, and higher concentrations were associated with clinical outcome.^10^, ^11^, ^12^ Serum NfL levels have been reported to be elevated at baseline in patients with GBS compared to age-matched healthy controls and were associated with disease severity.^13^^,^^14^ However, GBS is a heterogeneous disease with a highly dynamic initial phase, in which patients may clinically rapidly deteriorate, stabilise, or improve. Nerve conduction studies can reveal features of both demyelination and axonal degeneration in GBS; but, the results of these assessments can change during the acute phase of the disease. Moreover, the specific dynamics of serum NfL throughout the clinical course of GBS are largely unknown.

Thus, in this study, we aimed to validate earlier findings regarding serum NfL levels in relation to disease severity, electrophysiological subtype, clinical course, and outcomes in patients with GBS. Moreover, using longitudinal data, we aimed to describe the dynamics of serum NfL and sought to evaluate the independent prognostic significance of serum NfL at different time points in the acute phase of the disease relative to established clinical prognostic factors.

## Methods

### Study design and participants

The patients with GBS included in this study participated in the double-blind, randomised, placebo-controlled phase 3 SID-GBS trial to investigate the effects of a second course of IVIg (SID; 0.4 g IVIg/kg for 5 days) on the clinical outcomes of patients with a poor prognosis.^15^^,^^16^ The mEGOS score^5^ was determined 7–9 days after the start of the first IVIg course and patients with a poor prognosis (mEGOS 6–12) were randomly assigned to receive either SID or a single course of IVIg and placebo. Patients with a good prognosis (mEGOS 0–5) received the standard single IVIg course and were not randomised but underwent the same follow-up and assessment of outcome parameters as the randomised patients. The inclusion and exclusion criteria for the SID-GBS trial have been previously described in detail.^16^ In this study, we excluded patients for whom at least one serum sample was not available. The reference values for the 833 healthy controls were published by the Neurochemistry Laboratory Amsterdam UMC.^17^ No formal sample size or power calculation was conducted in preparation of the study.

### Data collection

Patients underwent clinical assessments before (baseline) and one, two, four, eight, twelve, and 26 weeks after the start of the first course of IVIg treatment. The GBS disability score (GBS-DS), a scale based on mobility and ventilation that ranges from 0 (no disability) to 6 (death), and the Medical Research Council (MRC) sum score, which ranges from 0 (quadriplegic) to 60 (normal strength), were assessed at every visit as indicators of disease severity. Nerve conduction studies (NCS) were classified according to the Hadden criteria.^18^ Sex of the participants was self-reported.

### Laboratory procedures

Serum samples were collected from patients at specific time points, including baseline (before treatment) and at one, two, four, and twelve weeks after the start of the first course of IVIg and stored at −80 °C. For this analysis, serum NfL levels were measured using the NF-Light Advantage Kit (Quanterix; Billerica, MA, USA) on a single-molecule array technology (Simoa) HD-X Analyzer (Quanterix) following the manufacturer's instructions. The serum samples were diluted at a ratio of 1:4, and in cases where the measurements fell outside the calibration curve, samples were further diluted and re-tested to fall within the calibration curve. The inter-assay variation (intermediate precision) was 8.5% and intra-assay variability (repeatability) was 3.3% based on three concentrations of quality control samples. Reference values were obtained for samples from healthy controls without neurological disorders from cohort studies that were previously analysed in the same laboratory on the same platform. NfL values obtained from EDTA-plasma (n = 102) were converted to serum NfL using the following formula: serum NfL [pg/ml] = −0.33 + 1.11 ∗ plasma NfL [pg/mL].^19^

### Ethics

The SID-GBS trial is registered with the Netherlands Trial Register, NTR 2224/NL2107. Approval was obtained from the Medical Ethical Committee of all participating centres and all patients provided written informed consent.

### Statistics

Linear mixed-effects models were used to describe the changes in serum NfL levels over time, while accounting for correlations between repeated measurements for each patient. In the fixed-effects part, we allowed for a nonlinear effect of time using natural cubic splines with three degrees of freedom and boundary knots placed at the 5th and 95th percentiles of the follow-up time point. The internal knots were placed according to percentiles of follow-up time. Patients' characteristics, including age, MRC sum score, GBS-DS, NCS subtype, and the interaction of non-linear time with all fixed-effect parameters, were assessed. The appropriate fixed-effects structure was selected based on F-tests and likelihood ratio tests. In the random-effects structure, we included the random intercepts and slopes of serum NfL over time for each individual and natural cubic splines with two degrees of freedom were employed to allow for non-linear evolutions over time. The appropriate random-effects structure was selected based on likelihood ratio tests for nested models. The underlying assumptions, including normality and homogeneity of variance of residuals, and linearity of quantitative predictors was checked using QQ-plots and residual plots. Missing values for the MRC sum score (missing: 3.4%) and GBS-DS (missing: 3.6%) were imputed using the R package ‘mice’. Single imputation was employed and predictors for the imputation model were chosen based on clinical expertise and review of previous literature. Based on the distribution of serum NfL and the full models that incorporate restricted cubic splines or fractional polynomials, we logarithmically transformed the serum NfL values.

In subsequent analysis we examined both raw NfL levels and age-corrected *z*-scores. Age-corrected *z*-scores for NfL levels were obtained using the formula described by Vermunt et al.^17^ and the rationale for using this formula is described in Supplementary Fig. S1.

We employed multivariable logistic regression analysis with ability to walk unaided as the dependent variable to investigate the prognostic value of serum NfL while adjusting for the individual components of the mEGOS, an outcome score routinely used in the clinic which includes age, preceding diarrhoea, and MRC sum score measured one week after initiation of the first course of IVIg. The linearity assumption underlying logistic regression models was visually assessed. The odds ratio (OR) was derived from binomial logistic regression. The risk ratio (RR) was derived from modified Poisson regression using a sandwich variance estimator. We categorised serum NfL at week one (5–12 days), week two (12–21 days), and week four (21–35 days) for cross-sectional analysis. Model performance was expressed in terms of goodness-of-fit (pseudo-R^2^, referred to as R^2^),^20^ and discrimination (C-statistic).^21^ The C-statistic represents how well the model differentiates between patients with a low and high risk of the outcome. When the C-statistic is 0.5, the model performs no better than chance, and when it is 1, the model perfectly discriminates. Optimism-corrected model performance estimates and confidence intervals were derived using bootstrapping of the derivation dataset with resampling (*n* = 200). We performed a time-to-event analysis using the log-rank test to compare the cumulative incidence curves between groups stratified for NfL concentrations, considering time to regain the ability to walk unaided as the survival endpoint. The time-to-event was calculated from the start of the first IVIg treatment to the time the event occurred or the end of the study period.

Statistical analysis was performed using R statistical software (version 4.1.2). Spearman's rank correlation coefficient was computed for comparison of two variables, the Mann–Whitney U test for comparisons between two groups, and the Kruskal–Wallis test for comparisons between multiple groups. Mixed-effects models were fitted using the ‘lme’ function within the *nlme* package (version 3.1–160).^22^ The *GLMMadaptive* package (version 0.8.5)^23^ was used to construct effect plots. Logistic regression models were fitted using the *rms* package (version 6.6–0). p-values less than 0.05 were considered statistically significant.

### Role of funders

The SID-GBS trial was an investigator-initiated study funded by the Prinses Beatrix Spierfonds and Sanquin Plasma Products (Amsterdam, The Netherlands). The current project on serum NfL was sponsored by the Prinses Beatrix Spierfonds (W.OR19-24); the funders had no role in study design, data collection, data analysis, data interpretation, or writing of the manuscript.

## Results

### Baseline patient characteristics

Of the 327 patients enrolled to the SID-GBS trial, 13 were excluded before randomisation and six were excluded after randomisation. Of the remaining 308 patients, 295 fulfilled the criteria for the diagnosis of GBS and 281 of these patients had at least one serum sample available for analysis (Supplementary Fig. S2). The excluded patients did not differ from the remaining patients regarding demographic and baseline characteristics (data not shown). The patients had a mean age of 55 years (SD = 17) and 184 (65%) were male. Baseline characteristics are described in Supplementary Table S1. The median time from onset of first weakness to enrolment in the SID-GBS trial was 2 days (IQR = 1–4). The final dataset for this analysis included 192 patients who had a good prognosis after receiving one standard course of IVIg and were not randomised. The remaining 89 patients with a poor prognosis were randomised to receive either SID (*n* = 48) or placebo (*n* = 41). In total, 841 serum samples collected at baseline (prior to IVIg treatment) or during follow-up (range, 0–179 days) were analysed. The median number of follow-up samples per patient was three (range, 1–5).

The pre-treatment serum NfL levels of the patients with GBS were significantly higher compared to the reference values obtained from healthy controls (median = 27 pg/mL; IQR = 16–56 vs. 9 pg/mL, 6–12; p < 0.0001, Mann–Whitney U test), also when expressed as age-adjusted *z*-score compared with a reference database (Supplementary Table S2).^24^ In the patients with GBS, baseline serum NfL was significantly correlated with age (r = 0.37, 95% CI = 0.22–0.50, p < 0.0001, Spearman's rank test; Supplementary Fig. S3), but not with sex (p = 0.84, Mann–Whitney U test; Supplementary Fig. S4). The baseline serum NfL levels and *z*-scores were not significantly different between the groups of patients with the demyelinating, axonal, equivocal, inexcitable, and normal electrophysiological subtypes (p = 0.28 and p = 0.32, Kruskal–Wallis test; Supplementary Fig. S5) or the groups of patients with different GBS-DS at entry (p = 0.39 and p = 0.44, Kruskal–Wallis test; Supplementary Fig. S6a and b). However, a weak, yet significant, correlation was observed between serum NfL levels and MRC sum score at baseline (r = −0.18, 95% CI = −0.33 to −0.023, p = 0.026; Spearman's rank test, Supplementary Fig. S6c), but not when transformed to *z-*scores (r = −0.078, 95% CI = −0.24 to 0.084, p = 0.35, Spearman's rank test; Supplementary Fig. S6d).

### Longitudinal pattern of NfL levels and stratifications

**Fig. 1:**
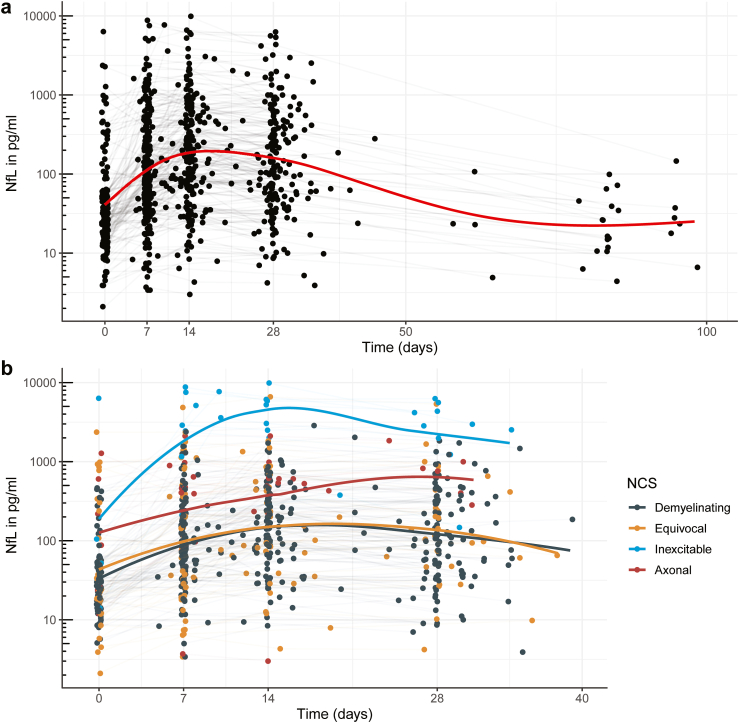
Serum NfL levels during the course of disease in patients with GBS. a) Evolution of serum NfL in patients with GBS. A jitter of ±0.5 days was allowed. Two samples taken after 100 days were omitted. The red line represents the locally weighted scatterplot smoothing (LOESS) curve. b) Similar to a), but colours represent the NCS subtype. Samples taken after 40 days were omitted. Data for ‘not assessed’ and ‘normal’ NCS variants are not shown in this figure. Grey lines connect samples from the same patient. *Abbreviations:* NCS, Nerve conduction studies; NfL, Neurofilament light chain.

In general, in patients with GBS, serum NfL continued to increase between baseline and one and two weeks after initiation of treatment, remained high at one month, and decreased by three months (Fig. 1a). The variability in the course of serum NfL was considerable and varied by a factor of 1000 between patients at every time point. Furthermore, serum NfL at week 1 and subsequent follow-up time points was highly associated with the NCS subtype, as patients with the inexcitable and axonal subtypes had higher serum NfL levels compared to the subgroups with the demyelinating or equivocal subtypes (Fig. 1b). However, it is important to note that NfL was also elevated in the majority of patients with the demyelinating form of GBS.

**Fig. 2:**
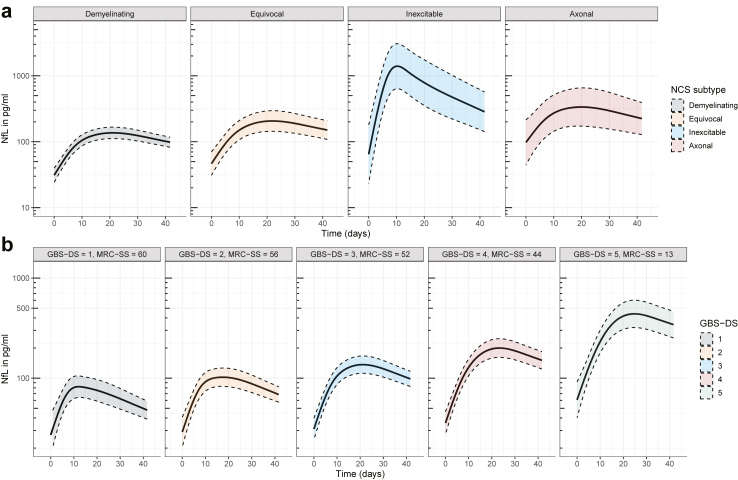
Mixed-effects models for the evolution of serum NfL in patients with GBS. a) Estimated effect of NCS subtype on the dynamics of NfL. Age, MRC-SS, and GBS-DS were set on the median. b) Estimated effect of disease severity on the dynamics of NfL. Age was set on the median; NCS subtype, on ‘demyelinating’. Continuous lines represent the estimated NfL levels; the dashed lines represent the 95% confidence interval of the estimate. *Abbreviations:* NCS, Nerve conduction studies; NfL, Neurofilament light chain; MRC-SS, Medical Research Council sum score; GBS-DS, Guillain-Barré syndrome disability score.

A mixed-effects model of log (NfL) over time indicated a significant interaction between the NCS subtype and the dynamics of serum NfL (likelihood-ratio test p < 0.0001, Fig. 2a). Patients with the inexcitable subtype showed a sharp rise in serum NfL that peaked around 10 days. Conversely, patients with the demyelinating, axonal, and equivocal subtypes exhibited peak serum NfL around 20 days. The subgroups with the inexcitable subtype or the axonal NCS subtype exhibited higher serum NfL levels throughout the course of disease compared to patients with a demyelinating or equivocal NCS subtype.

A significant interaction was observed between the GBS-DS and time on serum NfL levels (likelihood-ratio test p < 0.0001), indicating that patients with more severe disease exhibited a sharper increase in serum NfL and subsequently slower normalisation towards baseline serum NfL levels. More severe limb weakness, as indicated by a lower MRC sum score, correlated with higher serum NfL (*b* = −0.017, 95% CI = −0.024 to −0.011), but had no significant association with the dynamics of serum NfL. A positive correlation was observed between disease severity and serum NfL, particularly for patients with the demyelinating NCS subtype (Fig. 2b). The association between the GBS-DS and serum NfL was less apparent for other NCS subtypes (Supplementary Fig. S7). Diagnostic plots showed that all the model assumptions were valid.

### Prognostic value and association between serum NfL and clinical outcomes

Serum log (NfL) at two weeks after the start of treatment was significantly associated with inability to walk unaided at four weeks, independently of the individual components of the mEGOS (OR = 1.74, 95% CI = 1.27–2.45; Table 1); serum log (NfL) at baseline, one week, and four weeks were also significantly associated with inability to walk unaided at four weeks (Table 1). Similarly, NfL measured at every time point, except week 1, was significantly associated with the risk of inability to walk unaided (Table 1). The highest association between inability to walk unaided at 26 weeks and serum log (NfL) was observed at four weeks (OR = 2.79, 95% CI = 1.72–4.90, Table 1); serum log (NfL) at baseline, one week, and two weeks were also significantly associated with the inability to walk unaided at 26 weeks, also when expressed as RRs (Table 1). Similar results were found using age corrected *z*-scores (Supplementary Table S3). To determine the robustness of our imputation approach, we performed the same analysis on observed-only data and found similar results (Supplementary Table S4).

**Table 1:** Multivariable regression analysis of the associations of serum NfL with the modified Erasmus GBS outcome score (mEGOS) and inability to walk unaided at 4 and 26 weeks.

Multivariable logistic regression	Inability to walk unaided at 4 weeks				Inability to walk unaided at 26 weeks			
	N (unable)	OR (95% CI)	p	RR (95% CI)	N (unable)	OR (95% CI)	p	RR (95% CI)
Log (NfL) entry	138 (67)	1.71 (1.21–2.54)	0.0039	1.16 (1.03–1.30)	137 (16)	2.25 (1.45–3.74)	0.00063	1.48 (1.18–1.86)
Age, years		1.02 (0.99–1.05)	0.13	1.01 (1.00–1.02)		1.01 (0.96–1.05)	0.75	1.01 (0.98–1.05)
Preceding diarrhoea		0.60 (0.21–1.58)	0.31	0.81 (0.58–1.13)		0.59 (0.13–2.28)	0.47	0.62 (0.22–1.72)
MRC-SS week 1		0.89 (0.84–0.93)	<0.0001	0.97 (0.97–0.98)		0.93 (0.89–0.96)	0.00029	0.96 (0.94–0.98)
Log (NfL) week 1	243 (123)	1.40 (1.08–1.83)	0.013	1.07 (0.99–1.15)	242 (30)	1.59 (1.15–2.26)	0.0067	1.30 (1.01–1.68)
Age, years		1.03 (1.00–1.05)	0.018	1.01 (1.00–1.02)		1.03 (1.00–1.06)	0.10	1.02 (0.99–1.04)
Preceding diarrhoea		1.08 (0.52–2.25)	0.83	1.02 (0.80–1.29)		1.08 (0.38–2.80)	0.90	0.99 (0.56–1.75)
MRC-SS week 1		0.88 (0.84–0.91)	<0.0001	0.98 (0.97–0.98)		0.95 (0.92–0.97)	<0.0001	0.96 (0.95–0.98)
Log (NfL) week 2	213 (116)	1.74 (1.27–2.45)	0.00086	1.14 (1.05–1.25)	206 (29)	1.76 (1.18–2.75)	0.0084	1.38 (1.04–1.83)
Age, years		1.02 (1.00–1.05)	0.084	1.01 (1.00–1.02)		1.03 (0.99–1.06)	0.14	1.02 (0.99–1.05)
Preceding diarrhoea		1.17 (0.51–2.70)	0.70	0.99 (0.79–1.24)		0.91 (0.30–2.54)	0.86	0.88 (0.52–1.50)
MRC-SS week 1		0.88 (0.84–0.92)	<0.0001	0.98 (0.98–0.99)		0.95 (0.92–0.98)	0.0003	0.97 (0.95–0.99)
Log (NfL) week 4	189 (100)	1.59 (1.16–2.22)	0.0044	1.16 (1.05–1.29)	185 (27)	2.79 (1.72–4.90)	<0.0001	1.95 (1.36–2.79)
Age, years		1.01 (0.99–1.04)	0.32	1.01 (1.00–1.01)		1.02 (0.98–1.06)	0.34	1.01 (0.98–1.04)
Preceding diarrhoea		0.76 (0.33–1.72)	0.52	0.89 (0.69–1.14)		0.93 (0.29–2.76)	0.90	0.82 (0.49–1.36)
MRC-SS week 1		0.90 (0.86–0.93)	<0.0001	0.98 (0.98–0.99)		0.97 (0.94–1.00)	0.065	0.99 (0.97–1.00)

Numbers of patients unable to walk are indicated in brackets. Predictor variables with <15% missing values were imputed. NfL levels and missing values in the outcome variables were not imputed. The OR and RR correspond to a one-unit increase in the predictor. The p-value is derived from the binomial logistic regression. The RR is calculated from the modified Poisson regression using a sandwich variance estimator. CI, confidence interval; MRC-SS, Medical Research Council sum score; NfL, neurofilament light chain; OR, odds ratio; RR, risk ratio.

In logistic regression analysis of the binary outcome ability to walk unaided at four weeks, the reference models including the individual components of the mEGOS, without serum log (NfL), discriminated very well with optimism-corrected C-statistics ranging from 0.822 (95% CI = 0.747–0.886) to 0.880 (95% CI = 0.831–0.926; Table 2). Addition of serum log (NfL) at baseline contributed to the discriminative capability of the model, leading to an improvement in the C-statistic of 0.031 (95% CI = 0.001–0.081) and a clear trend towards improved performance (delta R^2^ = 5.9%, 95% CI = −0.2 to 15.9). Addition of serum log (NfL) at the other time points improved the C-statistic and R^2^ to a lesser extent (Table 2). In logistic regression analysis of the outcome ability to walk unaided at 26 weeks, the C-statistics for the reference models ranged from 0.727 (95% CI = 0.613–0.837) to 0.819 (95% CI = 0.736–0.894; Table 2). The improvement in the C-statistic ranged from 0.060 (95% CI = 0.027–0.101) after addition of serum log (NfL) at week four to the model to 0.109 (95% CI = 0.008–0.221) when serum NfL at entry was added to the model. Model performance improved by 12.6% R^2^ (95% CI = 7.4–21.8) after addition of log (NfL) at week four and by 14.9% R^2^ (95% CI = 0.6–33.1) when log (NfL) at entry was included (Table 2). Similar results were found using age-corrected *z*-scores (Supplementary Table S5).

**Table 2:** Optimism-corrected discriminative ability (C-statistic) of serum log (NfL) measured at different time points adjusted for individual components of the mEGOS.

Outcome: Inability to walk unaided at 4 weeks						
Time	Reference model		Model with log (NfL)		Delta C-statistic	Delta R^2^%
	C-statistic	R^2^%	C-statistic	R^2^%		
Entry	0.822 (0.747–0.886)	40.1 (24.2–54.7)	0.853 (0.780–0.903)	46.0 (30.7–58.8)	0.031 (0.001–0.081)	5.9 (−0.2 to 15.9)
Week 1	0.861 (0.811–0.897)	48.7 (36.5–59.7)	0.864 (0.810–0.899)	50.8 (38.3–61.2)	0.004 (−0.006 to 0.020)	2.0 (−0.2 to 6.1)
Week 2	0.880 (0.831–0.926)	52.4 (40.5–66.8)	0.891 (0.842–0.929)	56.3 (44.2–68.0)	0.011 (−0.006 to 0.035)	3.9 (−0.5 to 10.5)
Week 4	0.845 (0.791–0.893)	46.1 (32.7–57.9)	0.858 (0.808–0.903)	49.6 (37.8–61.1)	0.012 (−0.005 to 0.039)	3.5 (−0.6 to 9.5)
**Outcome: Inability to walk unaided at 26 weeks**						

**Time**	**Reference model**		**Model with log(NfL)**		**Delta C-statistic**	**Delta R**^**2**^**%**
	**C-statistic**	**R**^**2**^**%**	**C-statistic**	**R**^**2**^**%**		
Entry	0.727 (0.613–0.837)	15.2 (0–35.1)	0.836 (0.721–0.915)	30.2 (8.6–51.3)	0.109 (0.008–0.221)	14.9 (0.6–33.1)
Week 1	0.813 (0.736–0.879)	29.1 (13.8–43.8)	0.820 (0.740–0.887)	33.5 (16.5–47.3)	0.007 (−0.020 to 0.048)	4.3 (−1.0 to 14.2)
Week 2	0.819 (0.736–0.894)	32.0 (16.6–48.7)	0.832 (0.743–0.909)	35.7 (17.9–53.1)	0.012 (−0.015 to 0.053)	3.7 (−1.7 to 12.9)
Week 4	0.792 (0.710–0.865)	28.3 (12.9–46.1)	0.851 (0.770–0.928)	40.9 (23.6–58.3)	0.060 (0.027–0.101)	12.6 (7.4–21.8)

Data in parentheses represent the 95% confidence intervals. Time represents NfL measurement at entry or after initiation of the first course of IVIg. The reference model includes: age, preceding diarrhoea, and MRC sum score at week 1. Delta C-statistic and Delta R^2^ represent the differences between the model with log (NfL) and the reference model. Abbreviation: NfL, Neurofilament light chain.

**Fig. 3:**
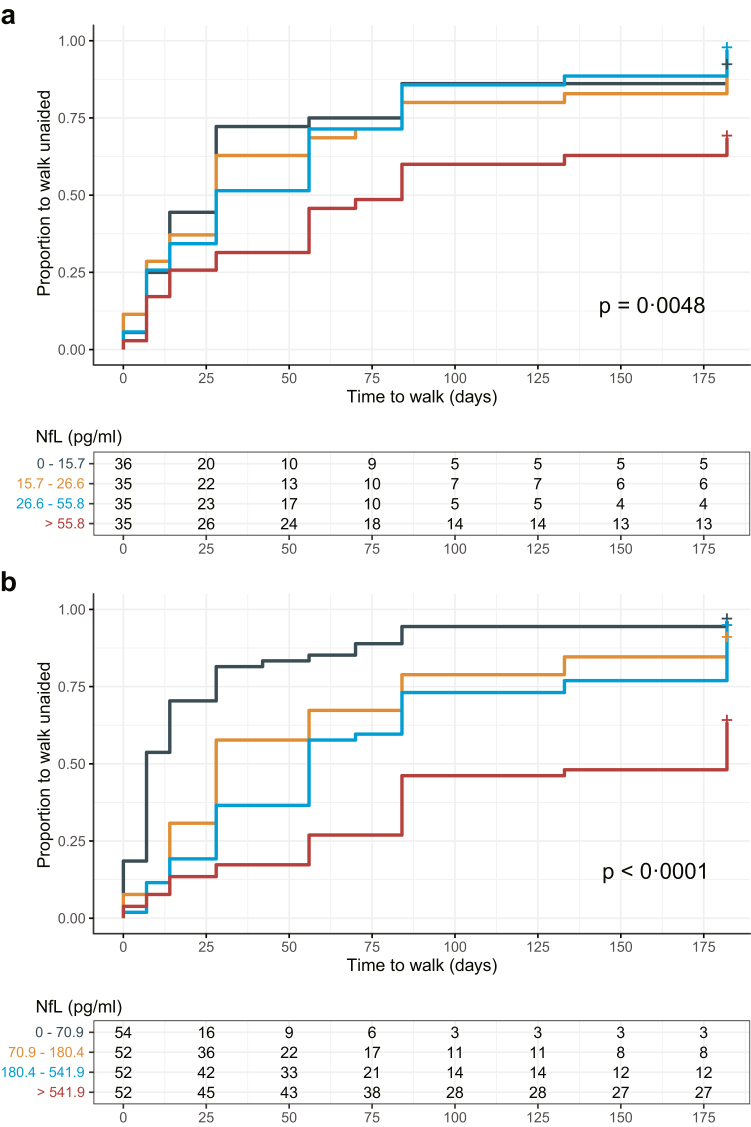
Time-to-event analysis for the ability to walk unaided in patients with GBS. Patients were stratified into quartiles based on their serum NfL levels at two visits: study entry (a), and week 2 (b). The p-values were obtained using the log-rank test. *Abbreviation:* NfL, Neurofilament light chain.

The subgroup of patients with the highest serum NfL levels at baseline required a significantly longer time to regain the ability to walk unaided, and a larger proportion of patients in this subgroup were unable to walk unaided at the end of the study (p = 0.0048, log-rank test; Fig. 3a). The quartile of patients with the highest serum NfL levels at two weeks regained the ability to walk unaided significantly later than other groups (p < 0.0001, log-rank test; Fig. 3b). Patients with an age-adjusted *z*-score >2.5, also took significantly longer to regain the ability to walk unaided (Supplementary Fig. S8).

### Association between serum NfL levels and treatment group

**Fig. 4:**
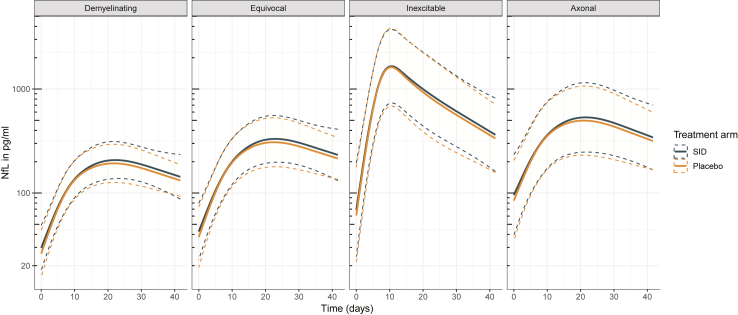
Estimated effect of treatment on serum NfL levels. Treatment group, placebo or a second IVIg course (SID), was included in the mixed-effects models (Model 2, Supplementary Material). Effect plots were stratified for NCS. Continuous lines represent the estimated NfL levels; the dashed lines represent the 95% confidence interval of the estimate. *Abbreviations:* NfL, Neurofilament light chain; NCS, Nerve conduction studies; SID, Second IVIg dose.

Finally, we investigated whether a second course of IVIg influenced the dynamics of serum NfL. In line with the lack of a clinical effect of a second IVIg course in the SID-GBS trial, addition of the treatment group to the mixed-effects model did not result in a difference between the patients who received standard IVIg treatment and placebo and the group who were randomised for a second course of IVIg (Fig. 4). Patients with serious adverse events generally had higher NfL levels, although this is confounded by more severe disease as most events occurred in the randomised patients (Supplementary Fig. S9).

## Discussion

This longitudinal study of patients enrolled in the SID-GBS trial demonstrates that distinct serum NfL dynamics are associated with the clinical course and electrophysiological subtype of GBS. In most patients with GBS, serum NfL was elevated early in the course of disease and continued to rise in the first two weeks, despite treatment with IVIg. High serum NfL levels from one week after initiation of IVIg and onwards were associated with axonal degeneration or inexcitable nerves in NCS, and with more severe weakness and disability, reflecting the presence and extent of axonal damage during the disease course. Importantly, in line with previous reports, we demonstrated that high serum NfL levels determined at entry and after the start of IVIg treatment are associated with a more severe clinical course and poorer outcome in GBS, independently of other prognostic factors. Moreover, serum NfL levels had added prognostic value to the mEGOS model for the outcome inability to walk unaided.

GBS typically exhibits a monophasic course; however, the dynamics of neuronal injury in individual patients are undefined.^25^ In accordance with previous studies,^13^^,^^14^^,^^26^ pre-treatment serum samples obtained shortly after the onset of weakness exhibited strongly elevated (3.5-fold-higher) NfL levels compared to age-specific reference values from healthy controls. The baseline NfL levels varied significantly within the group of patients with GBS, which indicates the extent of axonal damage varies between patients at hospital admission. We found that serum NfL continued to rise between baseline and one and two weeks in most patients and remained elevated for up to one month. Serum NfL usually did not normalise to healthy control levels within the study period of twelve weeks, although serum NfL was only measured in a small proportion (*n* = 25) of patients at twelve weeks. A previous study demonstrated that serum NfL levels of patients with GBS returned to similar levels as healthy controls at one year of follow-up,^14^ which is consistent with the monophasic disease course of GBS. Similar findings were observed in patients with traumatic brain injury, in whom serum NfL was elevated immediately after admission to the neuro-intensive care unit, continued to rise by twelve days post-injury, and then normalised by one year of follow-up.^27^ The observed dynamics of serum NfL seem to reflect the typical monophasic clinical disease course of GBS.

One important question to address is whether longitudinal measurements of serum NfL can be used to monitor the process of axonal damage as a measure of ongoing disease activity. In this study, we observed that during the acute phase of disease, from one week onwards, elevations in serum NfL were strongly correlated with the clinical course. Specifically, serum NfL increased by an average of almost 2-fold between one and two weeks in the majority of patients (>70%) and continued to increase between two and four weeks in approximately one third of patients. These continued increases in serum NfL suggest that ongoing axonal damage occurs in a subset of patients with GBS. One possible explanation could be that anti-ganglioside antibodies may persist and contribute to continuation of the damage to neuronal membranes.^28^ Alternatively, NfL may be released both in the later stages of axonal damage and early phases of repair and recovery.

In contrast to previous studies, we found no association between baseline serum NfL and disease severity as defined by the GBS-DS.^13^^,^^14^ One possible explanation is that most of the baseline samples tested in the our study were collected within two days after the onset of symptoms, compared to a median of four days in other related studies.^13^^,^^14^ As a consequence, we observed lower baseline serum NfL levels than previously reported in patients with GBS. Overall, our findings indicate that serum NfL does not represent a biomarker of disease severity in this very early stage of the disease.

Acute inflammatory demyelinating polyneuropathy (AIDP) and acute motor axonal neuropathy (AMAN) were proposed as two distinct subtypes of GBS^29^; however, at present, there is no consensus on the electrophysiological criteria or best timing of NCS to differentiate between these subtypes.^30^ The high levels of NfL in patients with inexcitable nerves suggest the occurrence of axonal damage more than reversible conduction block, at least in this study cohort. Nevertheless, most patients classified as demyelinating or equivocal in the current study also exhibited high serum NfL in the first 2 weeks indicating that to some extent axonal involvement occurs in most—if not all—patients with GBS.

Consistent with the results of the clinical trial, longitudinal analysis of the samples obtained from patients who received one vs. two standard courses of IVIg showed that the second course of treatment did not prevent a further rise in serum NfL, which likely further indicates that a continuous process of axonal degeneration occurs in GBS, despite treatment with IVIg. Nevertheless, the strong correlations between serum NfL and the disability scores during the full course of the disease may suggest that longitudinal monitoring of serum NfL could hold potential as an objective (intermediate) outcome measure to evaluate new treatments for GBS.

Accurate prediction of the clinical course of GBS is crucial to provide tailored supportive care and effectively inform patients and their families. We found that addition of serum NfL levels consistently improved the performance of the currently established prognostic model, which is based on clinical characteristics, to predict the inability to walk unaided at four and 26 weeks. Notably, the most substantial improvement was observed when NfL at entry was added to the logistic regression model. Interestingly, we did not find a direct association between NfL levels at study entry and disease severity or electrophysiological subtype, which may initially seem counterintuitive. However, it is plausible that some patients experience limb weakness due to conduction block or demyelination, without having significant axonal damage. Despite their initial disability, these patients may ultimately have a more favourable outcome, and low serum NfL at baseline may prove to be an effective marker to further identify this subgroup of patients.^31^

This study has several limitations. First, due to the protocol of the RCT, insufficient data were available between four and twelve weeks, the period in which serum NfL may start to normalise; analysis of samples from this period in studies with more frequent sampling could be used to further specify the duration of the active stage of disease. However, we measured NfL at three time points during the therapeutic window for immunomodulatory treatments in GBS, which is probably the first four weeks after onset of disease. Moreover, despite the strong associations between serum NfL and the clinical course of the disease and electrophysiological subtypes at a group level, it remains to be established if serum NfL can be used as a marker of disease activity or prognosis in individual patients. This may be especially relevant given the large variability in the dynamics of serum NfL between patients. The sample size for the axonal and inexcitable subgroups were small, and studies in larger groups are required to confirm our findings. To determine whether NfL can be implemented as a prognostic marker for clinical decision making, regression based models with NfL need to be calibrated and externally validated on larger sample sizes.

Correction for confounders is essential for the accurate interpretation of biomarker levels in patients. Well-established associations were previously reported for serum NfL levels and age, body-mass index, renal function, and diabetes mellitus.^17^^,^^32^ Data on BMI, renal function, and diabetes mellitus were not available in our cohort, which should be regarded as a limitation of the current study. Additionally, NfL may be expressed in trace amounts in non-neuronal cells, including immune cells and Schwann cells.^33^ However, the contribution of these non-neuronal sources of NfL is probably minor compared to NfL released from damaged axons. The location of axonal damage is difficult to establish based on serum NfL levels only. The fact that NfL levels are increased in CSF early in the disease course, suggests at least proximal axonal damage at the nerve roots.^34^ The ratio between CSF and serum NfL may help to elucidate whether axonal damage is proximal and/or distal.^35^ Recently the intermediate filament peripherin, which is almost exclusively expressed in the peripheral nervous system has been proposed as a biomarker of disease activity in neurology.^36^ The incorporation of these biomarkers, along with longitudinal serum NfL measurements and the correction for established confounders, has the potential to increase our understanding of the origin of axonal damage in peripheral neuropathies and may ultimately lead to better prognostic models.

In conclusion, serum NfL is elevated at the start of treatment in the majority of patients with GBS and continues to rise during the progressive phase of disease. Dynamics of serum NfL levels are associated with disease severity, and potentially may serve as a prognostic and predictive biomarker for clinical outcome and treatment response in patients with GBS. This study, in line with previous research, indicates a distinctive role for NfL in GBS.

## Contributors

ST, CT, RH, and BJ conceptualised the study. ST and CW curated the data. ST, CT, CM, RT, CW, HH, and BJ analysed the data. PD and BJ acquired funding. ST, CT, CM, RT, CW, HH, RH, PD, and BJ took part in the investigation. ST, CM, CW, and BJ created the methodology. ST, CT, RH, PD, and BJ took part in the project administration. ST, CT, CM, RT, CW, HH, RH, PD, and BJ provided resources. ST and CM provided the software. CT, RH, PD, and BJ gave supervision. ST, CT, CM, RH, PD, and BJ provided validation. ST and CM created the figures visualisation. ST and BJ wrote the original draft. All authors read and approved the final version of the manuscript. ST and BJ had access to raw data and final responsibility for the decision to submit for publication.

## Data sharing statement

In compliance with the General Data Protection Regulation, the source data cannot be made available to other researchers as patient approval has not been obtained for sharing coded data. Information about the analytic methods, syntax, and output files of statistical analyses will be made available by the corresponding author upon reasonable request.

## Declaration of interests

Research of CT is supported by the European Commission (Marie Curie International Training Network, grant agreement No 860197 (MIRIADE), Innovative Medicines Initiatives 3TR (Horizon 2020, grant no 831434) EPND (IMI 2 Joint Undertaking (JU), grant No. 101034344) and JPND (bPRIDE), National MS Society (Progressive MS alliance), Alzheimer Drug Discovery Foundation, Alzheimer Association, Health Holland, the Dutch Research Council (ZonMW), The Selfridges Group Foundation, Alzheimer Netherlands. CT is recipient of ABOARD, which is a public-private partnership receiving funding from ZonMW2 (#73305095007) and Health ∼ Holland, Topsector Life Sciences & Health (PPP-allowance; #LSHM20106). CT is recipient of TAP-dementia, a ZonMw funded project (#10510032120003) in the context of the Dutch National Dementia Strategy. CT performed contract research for ADx Neurosciences, AC-Immune, Aribio, Axon Neurosciences, Beckman–Coulter, BioConnect, Bioorchestra, Brainstorm Therapeutics, Celgene, Cognition Therapeutics, EIP Pharma, Eisai, Eli Lilly Fujirebio, Grifols, Instant Nano Biosensors, Merck, Novo Nordisk, Olink, PeopleBio, Quanterix, Roche, Siemens, Toyama, and Vivoryon. CT received payment or honoraria for lectures, presentations or educational events from Roche, Novo Nordisk and Grifols. All payments were made to her institution. CT serves on editorial boards of Medidact Neurologie/Springer; and in Neurology: Neuroimmunology & Neuroinflammation. She is editor of Alzheimer Research and Therapy. RH reports institutional funding from the GBS-CIDP Foundation International, Stichting GBS, T2B collaboration project funded by PPP Allowance made available by Top Sector Life Sciences & Health to Samenwerkende Gezondheidsfondsen (SGF) under project number LSHM18055-SGF to stimulate public-private partnerships and co-financing by health foundations that are part of the SGF, NIH, and PANDIA collaboration project co-funded by BÜHLMANN Laboratories AG and the PPP Allowance made available by Health ∼ Holland, Top Sector Life Sciences & Health, to Prinses Beatrix Spierfonds to stimulate public-private partnerships under project number LSHM23017-SGF. She serves as editorial board member of the Journal of the Peripheral Nervous System and was board member of the Inflammatory Neuropathy Consortium. PD reports grants from the Prinses Beatrix Spierfonds and Sanquin to conduct the SID-GBS trial. PD reports consulting fees from Annexon and Roche, all paid to the institution. PD participates on a Data Safety Monitoring Board or Advisory Board for Argenx, Hansa, Octapharma and Sanofi. All fees go to the institution. He reports an unpaid leadership role in the Peripheral Nerve Society (PNS) and serves on the Medical advisory board for the GBS/CIDP Foundation International. BJ reports institutional funding from the GBS-CIDP Foundation International, Stichting GBS, Horizon 2020 (EU), Grifols, CSL-Behring, Annexon, Hoffmann-la Roche, Hansa Biopharma, BÜHLMANN Laboratories. BJ received institutional royalties, licences or consulting fees from Hansa Biopharma, Annexon and Hoffmann-la Roche. BJ received support from the GBS-CIDP Foundation International to cover travel expenses for their conference. BJ serves an unpaid role in the global Medical advisory board for the GBS-CIDP Foundation International, in the scientific review committee of Stichting MS research, and the scientific review committee of the Erasmus MC. All other authors declare no competing interests.
