# Supplementary material for: Dynamics and prognostic value of serum neurofilament light chain in Guillain-Barré syndrome
Source: eBioMedicine. 2024 Mar 22;102:105072. doi: 10.1016/j.ebiom.2024.105072 (PMC10980997; doi:10.1016/j.ebiom.2024.105072)
Supplement: Supplementary Material [file mmc1.docx]

**Supplementary appendix**

**Contents Page**

Supplementary Statistical analysis 2

Supplementary Tables

Table S1: Baseline characteristics 3

Table S2: Age-corrected *z*-scores for NfL at entry 4

Table S3: Multivariable regression analysis of age-corrected *z*-scores 5

Table S4: Multivariable regression analysis of age-corrected *z*-scores (complete data) 6

Table S5: Discriminative ability of age-adjusted *z-*scores corrected for predictors mEGOS 7

Supplementary Figures

Figure S1: *z*-scores versus raw NfL concentrations in the patient cohort 8

Figure S2: Flowchart for selection of the patient cohort 9

Figure S3: Correlation between age and serum NfL at baseline 10

Figure S4: Correlation between NfL z-scores and sex in patients with GBS 11

Figure S5: Correlation between serum NfL at baseline (study entry) or age-corrected *z-*scores and NCS subtypes in GBS 12

Figure S6: Correlation between serum NfL at baseline (study entry) or age-corrected *z-*scores and disease severity 13

Figure S7: Evolution of serum NfL over time in patients with different NCS variants 14

Figure S8: Time-to-event analysis stratified on age-adjusted *z*-scores for NfL 15

Figure S9: Association between adverse events and NfL levels 16

References 17

**SUPPLEMENTARY STATISTICAL ANALYSIS**

All analyses were performed in R using the *nlme* package (https://cran.r-project.org/web/packages/nlme/nlme.pdf).

**Model 1:** NfL mixed effect model (Figure 2):
lme(log(NfL) ~ ns(time, df = 3, B = c(0, 816)) * (GBS-DS + NCS) + MRC sum score + age,
 random = ~ ns(time, df = 2, B = c(0, 816)) | ID)

**Model 2:** NfL mixed effect model + treatment group (Figure 4):
lme(log(NfL) ~ ns(time, df = 3, B = c(0, 816)) * (GBS-DS + NCS) + MRC sum score + age + treatment group,
 random = ~ ns(time, df = 2, B = c(0, 816)) | ID)

*Abbreviations:* B: boundary knots; ns: natural splines; df: degrees of freedom; ID: subject identifier

**SUPPLEMENTARY TABLES**

***Supplementary Table 1:* Baseline characteristics and serum NfL levels**

Overall baseline characteristics for the GBS patients and controls. *z*-scores are calculated based on the formula described by Vermunt et al.^1^ *Cross-sectional; ranges are described in the Materials and Methods. *Abbreviations:* IQR: Interquartile range; NfL: Neurofilament light chain; MRC: Medical Research Council; GBS: Guillain-Barré syndrome; URTI: Upper respiratory tract infection; NCS: Nerve conduction studies; mEGOS: Modified Erasmus GBS outcome score; N = Number of samples.

|  | **N** | **GBS**,  N = 281 | **N** | **Controls^1^** N = 833 |
| --- | --- | --- | --- | --- |
| **Sex, n (%)** | 281 |  | 833 |  |
| Male |  | 184 (65%) |  | 425 (51%) |
| Female |  | 97 (35%) |  | 408 (49%) |
| **Age years, Mean (SD)** | 281 | 55 (17) |  | 54 (13) |
| **Age years, range** |  | 14 – 87 |  | 19 – 85 |
| **GBS disability score at entry, N (%)** | 277 |  |  |  |
| 1 |  | 5 (2%) |  |  |
| 2 |  | 31 (11%) |  |  |
| 3 |  | 100 (36%) |  |  |
| 4 |  | 131 (47%) |  |  |
| 5 |  | 10 (4%) |  |  |
| **MRC sum score at entry, Median (IQR)** | 265 | 48 (44 – 52) |  |  |
| **Antecedent event, N (%)** | 281 |  |  |  |
| None |  | 82 (29%) |  |  |
| Diarrhoea |  | 76 (27%) |  |  |
| URTI |  | 72 (26%) |  |  |
| Other |  | 51 (18%) |  |  |
| **NCS variant, N (%)** | 276 |  |  |  |
| Demyelinating |  | 162 (59%) |  |  |
| Axonal |  | 14 (5%) |  |  |
| Equivocal |  | 52 (19%) |  |  |
| Inexcitable |  | 11 (4%) |  |  |
| Normal |  | 5 (2%) |  |  |
| Not performed |  | 29 (11%) |  |  |
| Not assessable |  | 3 (1%) |  |  |
| **mEGOS, Median (IQR)** | 275 | 4 (2-8) |  |  |
| **Samples per participant, Median (IQR)** | 281 | 3 (2-4) |  |  |
| **NfL pg/ml at entry*, Median (IQR)** | 148 | 27 (16 – 56) | 833 | 9 (6 – 12) |
| NfL, range |  | 2 – 6331 |  | 2 – 113 |
| ***z***-score, Median (IQR) |  | 2·4 (1·5 – 3·6) |  | 0·05 (-0.5 – 0.6) |
| **NfL pg/ml at week 1*, Median (IQR)**  NfL range | 256 | 97 (44 – 298)  3 – 8792 |  |  |
| *z*-score, Median (IQR) |  | 4·9 (3·3 – 7·4) |  |  |
| **NfL pg/ml at week 2*, Median (IQR)**  NfL, range | 219 | 180 (71 – 542)  3 – 9882 |  |  |
| *z*-score, Median (IQR) |  | 6·0 (4·1 – 8·3) |  |  |
| **NfL pg/ml at week 4*, Median (IQR)**  NfL, range | 194 | 127 (51 – 456)  4 – 6269 |  |  |
| *z*-score, Median (IQR) |  | 5·3 (3.4 – 8·1) |  |  |
| **NfL pg/ml at week 12*, Median (IQR)** |  | 18 (12 – 37) |  |  |
| NfL, range |  | 4 – 146 |  |  |
| *z*-score, Median (IQR) |  | 1·8 (1·1 – 2·7) |  |  |

***Supplementary Table 2:* Number of participants above the indicated age-corrected *z*-score in the control and the patient cohort.**

| Serum NfL  *z*-score (percentile) | Reference cohort Vermunt | | Reference cohort Benkert^2^ | | Normal distribution | Patient cohort  (at entry) | | Quotient:  PC (%) / RC (%) |
| --- | --- | --- | --- | --- | --- | --- | --- | --- |
|  | **n** | **%** | **n** | **%** | **% theor** | **n** | **%** |  |
| > 1 (84.13) | 117 | 14·0 | 712 | 15·7 | 15·9 | 124 | 83·8 | 6·0 |
| > 1.5 (93.32) | 57 | 6·8 | 291 | 6·4 | 6·7 | 115 | 77·7 | 11·4 |
| > 2 (97.72) | 27 | 3·2 | 102 | 2·3 | 2·3 | 92 | 62·2 | 19·4 |
| > 2.5 (99.40) | 13 | 1·6 | 30 | 0·7 | 0·6 | 75 | 50·7 | 31·7 |
| > 3 (99.87) | 3 | 0·4 | 11 | 0·2 | 0·1 | 38 | 25·7 | 64·3 |

A *z*-score of 1 corresponds to the 84.13^th^ percentile etc. All *z*-scores in this table were calculated from the formula described by Benkert et al. The reference control cohorts from Vermunt et al. and Benkert et al. were both similar and resembled the theoretical normal distribution. The quotient indicates the proportion of patients above the indicated z-score in the patient cohort divided by the proportion in the healthy control cohort. *Abbreviations:* NfL: Neurofilament light chain; PC: Patient cohort; RC: Reference cohort Vermunt; theor: theoretical.

***Supplementary Table 3.* Multivariable regression analysis of age-adjusted *z*-scores for NfL at different time points and individual components of the modified Erasmus GBS outcome score (mEGOS) in relation to inability to walk unaided at 4 and 26 weeks.**

| Multivariable logistic regression | Inability to walk unaided at 4 weeks | | | Inability to walk unaided at 26 weeks | | |
| --- | --- | --- | --- | --- | --- | --- |
|  | N (unable) | OR (95% CI) | P | N (unable) | OR (95% CI) | P |
| *z*-score NfL entry | 138 (67) | 1·29 (1·09-1·56) | 0·0047 | 137 (16) | 1·48 (1·20-1·89) | 0·00061 |
| Age, years |  | 1·04 (1·01-1·07) | 0·011 |  | 1·03 (0·99-1·08) | 0·20 |
| Preceding diarrhoea |  | 0·59 (0·21-1·55) | 0·29 |  | 0·59 (0·13-2·27) | 0·46 |
| MRC-SS week 1 |  | 0·89 (0·84-0·93) | < 0·0001 |  | 0·93 (0·89-0·96) | 0·00029 |
| *z*-score NfL week 1 | 243 (123) | 1·17 (1·03-1·33) | 0·019 | 242 (30) | 1·25 (1·07-1·48) | 0·0073 |
| Age, years |  | 1·04 (1·01-1·06) | 0·0016 |  | 1·04 (1·01-1·08) | 0·017 |
| Preceding diarrhoea |  | 1·08 (0·52-2·24) | 0·83 |  | 1·06 (0·38-2·80) | 0·90 |
| MRC-SS week 1 |  | 0·88 (0·84-0·91) | < 0·0001 |  | 0·95 (0·92-0·97) | < 0·0001 |
| *z*-score NfL week 2 | 213 (116) | 1·29 (1·11-1·52) | 0·0014 | 206 (29) | 1·30 (1·07-1·61) | 0·010 |
| Age, years |  | 1·04 (1·01-1·06) | 0·0037 |  | 1·04 (1·01-1·08) | 0·021 |
| Preceding diarrhoea |  | 1·17 (0·51-2·67) | 0·72 |  | 0·92 (0·31-2·56) | 0·87 |
| MRC-SS week 1 |  | 0·88 (0·84-0·92) | < 0·0001 |  | 0·95 (0·92-0·98) | 0·00021 |
| *z*-score NfL week 4 | 189 (100) | 1·24 (1·06-1·45) | 0·0068 | 185 (27) | 1·63 (1·29-2·14) | 0·00011 |
| Age, years |  | 1·02 (1·00-1·05) | 0·041 |  | 1·05 (1·01-1·10) | 0·021 |
| Preceding diarrhoea |  | 0·76 (0.33-1.71) | 0·51 |  | 0·94 (0·30-2·77) | 0·91 |
| MRC-SS week 1 |  | 0·90 (0.86-0.94) | < 0·0001 |  | 0·97 (0·94-1·00) | 0·059 |

Numbers of patients unable to walk are indicated in brackets. NfL levels and missing values in the outcome variables were not imputed. The OR corresponds to a one-unit increase in the predictor. *Abbreviations:* CI: Confidence interval; MRC-SS: Medical Research Council sum score; NfL: Neurofilament light chain; OR: Odds ratio.

***Supplementary Table 4.* Multivariable regression analysis of the associations of serum NfL with the modified Erasmus GBS outcome score (mEGOS) and inability to walk unaided at 4 and 26 weeks on complete data (no imputation) versus imputed data.**

| Multivariable logistic regression | Inability to walk unaided at 4 weeks | | | Inability to walk unaided at 26 weeks | | |
| --- | --- | --- | --- | --- | --- | --- |
|  | OR (95% CI) | P | OR (95% CI) | OR (95% CI) | P | OR (95% CI) |
| Log(NfL) entry | 1·71 (1·21-2·54) | 0·0039 | 1·73 (1·22-2·58) | 2·25 (1·45-3·74) | 0·00063 | 2·05 (1·31-3·44) |
| Age | 1·02 (0·99-1·05) | 0·13 | 1·02 (0·99-1·05) | 1·01 (0·96-1·05) | 0·75 | 1·01 (0·97-1·06) |
| Preceding diarrhoea | 0·60 (0·21-1·58) | 0·31 | 0·61 (0·22-1·62) | 0·59 (0·13-2·28) | 0·47 | 0·69 (0·15-2·70) |
| MRC-SS week 1 | 0·89 (0·84-0·93) | < 0·0001 | 0·89 (0·84-0·93) | 0·93 (0·89-0·96) | 0·00029 | 0·93 (0·89-0·96) |
| Log(NfL) week 1 | 1·40 (1·08-1·83) | 0·013 | 1·42 (1·09-1·87) | 1·59 (1·15-2·26) | 0·0067 | 1·58 (1·14-2·25) |
| Age | 1·03 (1·00-1·05) | 0·018 | 1·03 (1·01-1·05) | 1·03 (1·00-1·06) | 0·10 | 1·03 (1·00-1·06) |
| Preceding diarrhoea | 1·08 (0·52-2·25) | 0·83 | 1·11 (0·53-2·31) | 1·08 (0·38-2·80) | 0·90 | 1·07 (0·38 -2·83) |
| MRC-SS week 1 | 0·88 (0·84-0·91) | < 0·0001 | 0·88 (0·84-0·91) | 0·95 (0·92-0·97) | < 0·0001 | 0·95 (0·92-0·97) |
| Log(NfL) week 2 | 1·74 (1·27-2·45) | 0·0009 | 1·77 (1·28-2·50) | 1·76 (1·18-2·75) | 0·0084 | 1·82 (1·21-2·87) |
| Age | 1·02 (1·00-1·05) | 0·084 | 1·02 (1·00-1·05) | 1·03 (0·99-1·06) | 0·14 | 1·03 (0·99-1·07) |
| Preceding diarrhoea | 1·17 (0·51-2·70) | 0·70 | 1·21 (0·52-2·80) | 0·91 (0·30-2·54) | 0·86 | 0·88 (0·29-2·50) |
| MRC-SS week 1 | 0·88 (0·84-0·92) | < 0·0001 | 0·88 (0·84-0·92) | 0·95 (0·92-0·98) | 0·0003 | 0·95 (0·92-0·98) |
| Log(NfL) week 4 | 1·59 (1·16-2·22) | 0·0044 | 1·60 (1·17-2·23) | 2·79 (1·72-4·90) | < 0·0001 | 2·81 (1·73-4·92) |
| Age | 1·01 (0·99-1·04) | 0·32 | 1·01 (0·99-1·04) | 1·02 (0·98-1·06) | 0·34 | 1·02 (0·98-1·06) |
| Preceding diarrhoea | 0·76 (0.33-1.72) | 0·52 | 0·80 (0·35-1·79) | 0·93 (0·29-2·76) | 0·90 | 0·90 (0·28-2·67) |
| MRC-SS week 1 | 0·90 (0.86-0.93) | < 0·0001 | 0·90 (0·86-0·94) | 0·97 (0·94-1·00) | 0·065 | 0·97 (0·94-1·00) |

The OR corresponds to a one-unit increase in the predictor. *Abbreviations:* CI: Confidence interval; MRC-SS: Medical Research Council sum score; NfL: Neurofilament light chain; OR: Odds ratio.

***Supplementary Table 5.* Optimism-corrected discriminative ability (C-statistic) of age-adjusted NfL *z*-scores measured at different time points corrected for individual components of the mEGOS.**

| **Outcome: Inability to walk unaided at 4 weeks** | | | | | | |
| --- | --- | --- | --- | --- | --- | --- |
|  | **Reference model** | | **Model With *z*-score NfL** | |  |  |
| **Time** | **C-statistic** | **R^2^ %** | **C-statistic** | **R^2^ %** | **Delta C-statistic** | **Delta R^2^ %** |
| Entry | 0·822  (0·747 – 0·886) | 40·1  (24.2 –54.7) | 0·854  (0·781 – 0·904) | 45·6  (29·8 – 58·6) | 0·031  (0·004 – 0·072) | 5·5  (-0·4 – 15·5) |
| Week 1 | 0·861  (0·811 – 0·897) | 48.7  (36·5 –59·7) | 0·864  (0·813 – 0·900) | 50·5  (38·1 – 61·2) | 0·004  (-0·006 – 0·020) | 1·8  (-0·3 – 5·4) |
| Week 2 | 0·880  (0·831 – 0·926) | 52.4  (40·5 – 66·8) | 0·891  (0·842 – 0·930) | 55·9  (43·7 – 67·9) | 0·011  (-0·004 – 0·034) | 3·5  (-0·6 – 9·9) |
| Week 4 | 0·845  (0·791 – 0·893) | 46·1  (32·7 – 57·9) | 0·857  (0·807 – 0·901) | 49·2  (37·3 – 57·9) | 0·011  (-0·005 – 0·036) | 3·1  (-0·7 – 8·7) |
| **Outcome: Inability to walk unaided at 26 weeks** | | | | | | |
|  | **Reference model** | | **Model With *z*-score NfL** | |  |  |
| **Time** | **C-statistic** | **R^2^ %** | **C-statistic** | **R^2^ %** | **Delta C-statistic** | **Delta R^2^ %** |
| Entry | 0·727  (0·613 – 0·837) | 15·2  (-1·6 – 35·1) | 0·837  (0·728 – 0·912) | 30·1  (8·7 – 50·4) | 0·110  (0·010 – 0·218) | 14·9  (0·5 – 33·3) |
| Week 1 | 0·813  (0·736 – 0·879) | 29·1  (13·8 – 43·8) | 0·820  (0·741 – 0·887) | 33·3  (16·3 – 47·4) | 0·007  (-0·020 – 0·045) | 4·2  (-1·1 – 13·9) |
| Week 2 | 0·821  (0·741 – 0·896) | 32·4  (17·7 – 49·4) | 0·824  (0·740 – 0·902) | 34·2  (16·1 – 53·7) | 0·003  (-0·029 – 0·040) | 1·8  (-3·3 – 11·3) |
| Week 4 | 0·792  (0·710 – 0·881) | 28·3  (10·5 – 48·8) | 0·850  (0·765 – 0·924) | 40·6  (19·8 – 60·7) | 0·059  (0·003 – 0·132) | 12·3  (2·3 – 27·7) |

Data in parentheses represent the 95% confidence intervals. Time represents NfL measurement at entry or after initiation of the first course of IVIg. The reference model includes: age, preceding diarrhoea, and MRC sum score at week 1. Delta C-statistic and Delta-R^2^ represent the differences between the model with age-adjusted *z-*score and the reference model. *Abbreviations:* NfL: Neurofilament light chain.

**SUPPLEMENTARY FIGURES**

***Supplementary Figure S1*. *z*-scores versus NfL concentration**. a) Age-adjusted *z*-scores calculated from the formula by Vermunt et al.^1^ or derived from the model by Benkert et al.^2^ versus NfL concentrations in the patient population. The *z*-scores calculated with the formula by b) Vermunt et al. or by c) Benkert et al. stratified for age. The y-axis is on a log10 scale.


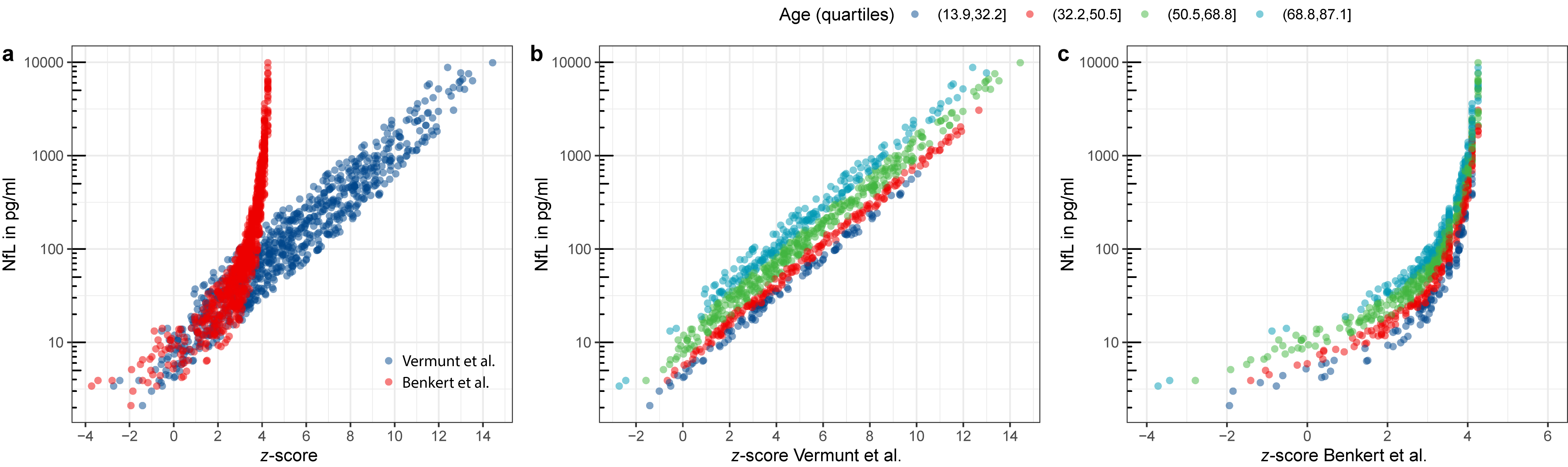


***Interpretation:*** *By definition, z-scores represent the number of standard deviations from a population mean. The z-scores obtained from Benkert et al. are tied to percentiles, resulting in a form of "compression" or data loss, as illustrated in the abovementioned figures. The formula proposed by Benkert et al. is undoubtedly crucial for identifying individuals at risk for a specific outcome and assessing treatment response, as elegantly demonstrated in the case of multiple sclerosis.^2^ However, using these continuous z-scores in subsequent analyses obscures relevant differences between patients with higher NfL levels within our GBS cohort, and reduces granularity of the data. Additionally, NfL concentrations for both the control cohort, as described in Vermunt et al., and our patient cohort were measured in the same laboratory. This consistency in data collection is highly relevant to ensure reliability of the data.*

***Supplementary Figure S2*. Flowchart for selection of the patient cohort**. *Other reasons include: transfer to a hospital abroad, pharmacy did not deliver treatment, second course received before randomisation, incorrect calculation of prognosis (*n* = 3), and case report files lost. *Abbreviations:* GBS: Guillain-Barré syndrome; SID: Second IVIg dose; CIDP: Chronic inflammatory demyelinating polyneuropathy.

339 assessed for eligibility

31 excluded
 9 did not meet inclusion criteria
 11 declined to participate
 2 lost to follow-up before primary endpoint
 2 did not receive allocated treatment
 7 other reasons*

308 included in final analysis SID-GBS trial

295 with diagnosis of GBS

13 excluded due to diagnosis of CIDP

281 included in analysis

14 excluded due to no serum available

***Supplementary Figure S3*. Correlation between age and serum NfL at baseline**. The reported P-value was obtained with Spearman’s rank correlation test. The shaded area represents the 95% confidence interval around the regression line. The y-axis is on a log10 scale. Coloured dots represent the GBS-DS at baseline. *Abbreviations:* NfL: Neurofilament light chain; GBS-DS: Guillain-Barré syndrome disability score.


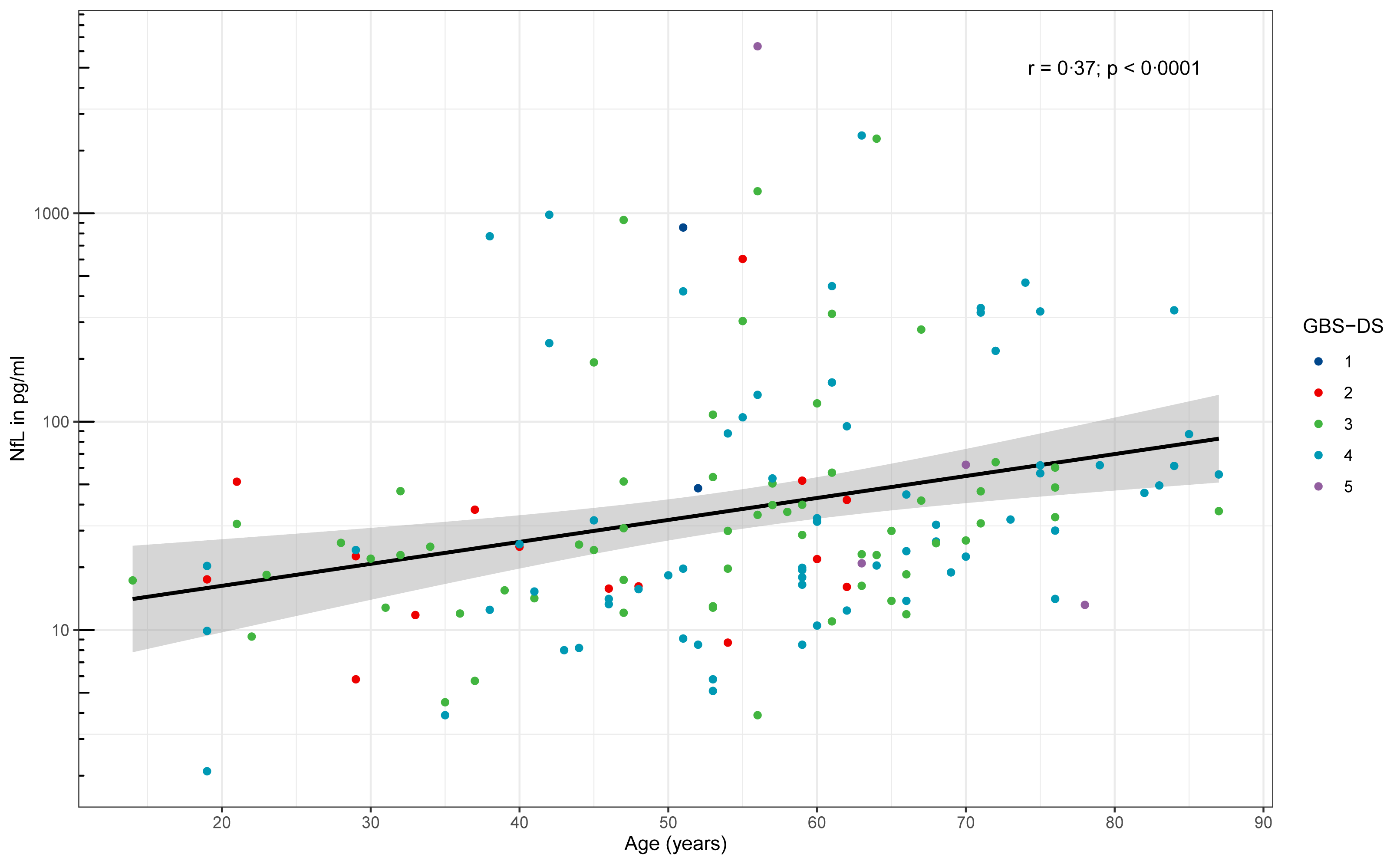


***Supplementary Figure S4*.** **Correlation between NfL *z-*scores and sex in patients with GBS stratified for time point.** Reported P-values were obtained using the Mann-Whitney U test. The box represents the IQR, and the whiskers show the range within 1.5*IQR. *Abbreviations:* NfL: Neurofilament light chain; IQR: Interquartile range.


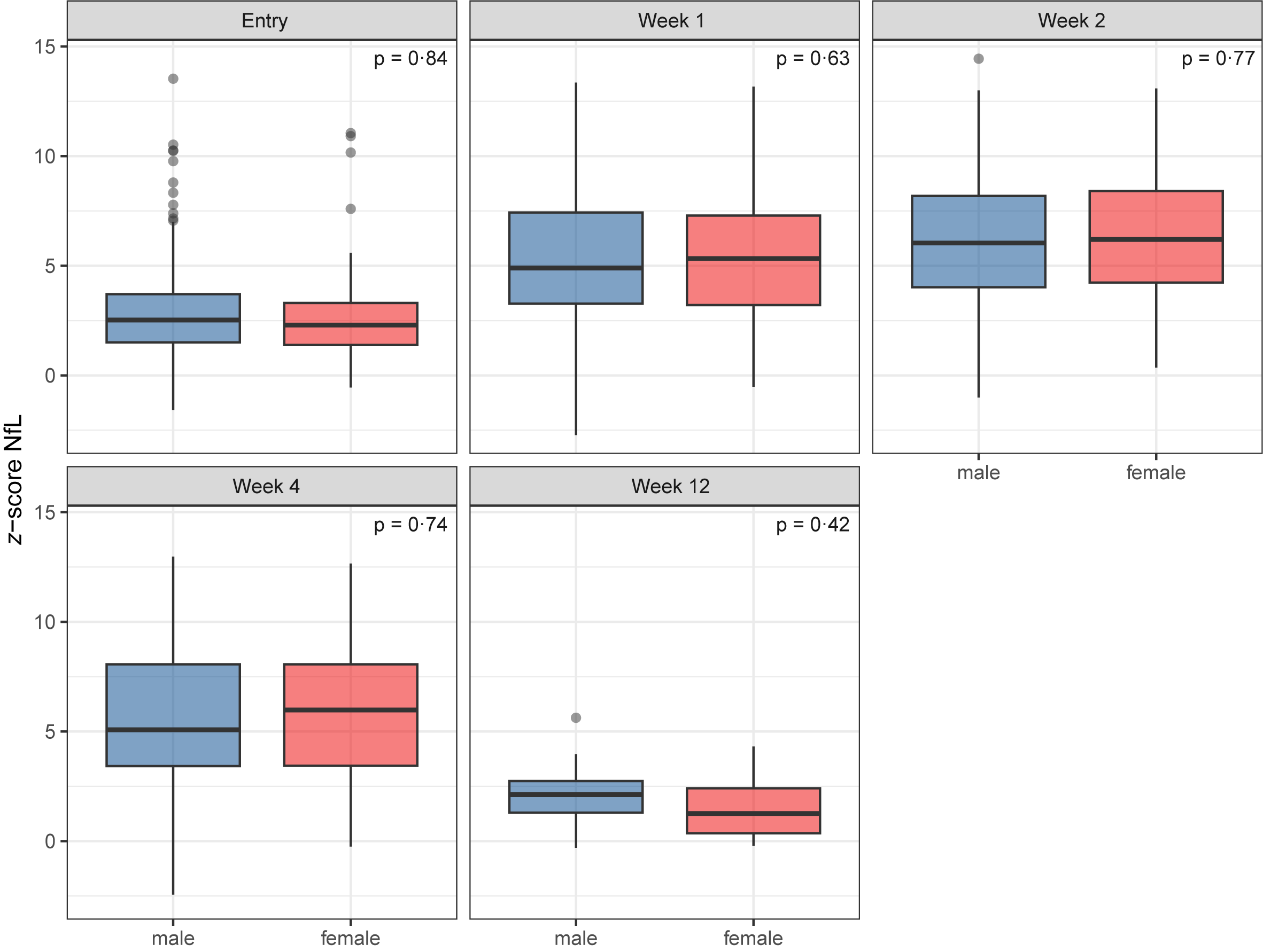


***Supplementary Figure S5*.** **Correlation between a) serum NfL at baseline (study entry) or b) age-corrected *z-*scores and NCS subtypes in GBS.** Reported P-values were obtained using the Kruskal-Wallis rank sum test. Data for patients for whom NCS was ‘not assessed’ (n = 11) or ‘not performed’ (n = 1) are not shown. In a), the y-axis is on a log10 scale. The box represents the IQR, and the whiskers show the range within 1.5*IQR. *Abbreviations:* NCS: Nerve conduction studies; IQR: Interquartile range.


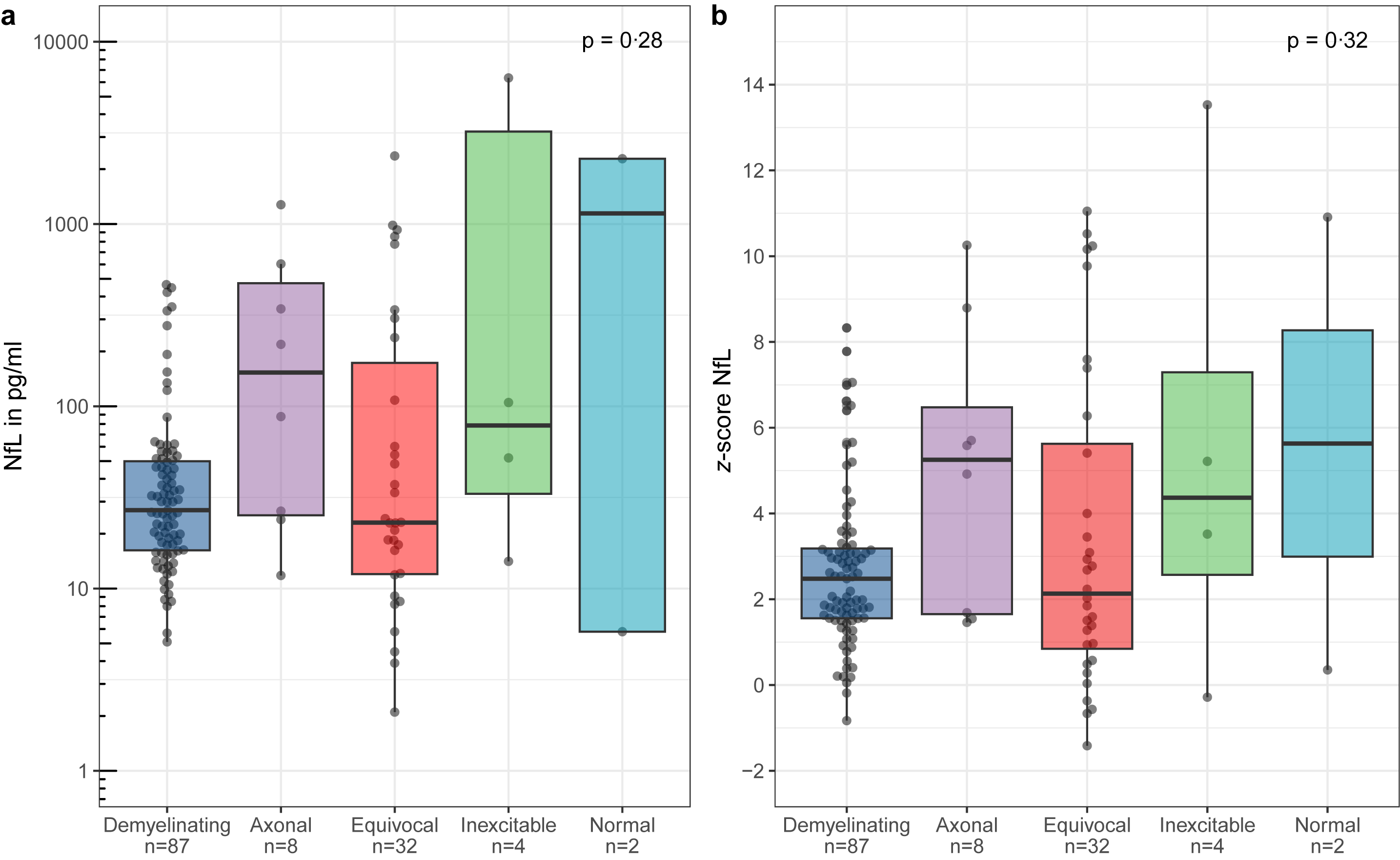


***Supplementary Figure S6*:** **Correlation between serum NfL at baseline (study entry) or age corrected *z-*score and disease severity**. The correlation between the GBS-DS at baseline and a) serum NfL or b) age-corrected *z*-scores, and the correlation between the MRC sum score at baseline and c) serum NfL or d) age-corrected *z*-scores. Reported P-values were obtained using the Kruskal-Wallis rank sum test for the GBS-DS and Spearman’s rank correlation test for the MRC sum score. The box (in a, b) represents the IQR, and the whiskers show the range within 1.5*IQR. The shaded area (in c, d) represents the 95% confidence interval around the regression line. *Abbreviations:* MRC: Medical Research Council; GBS-DS: Guillain-Barré syndrome disability score; IQR: Interquartile range.


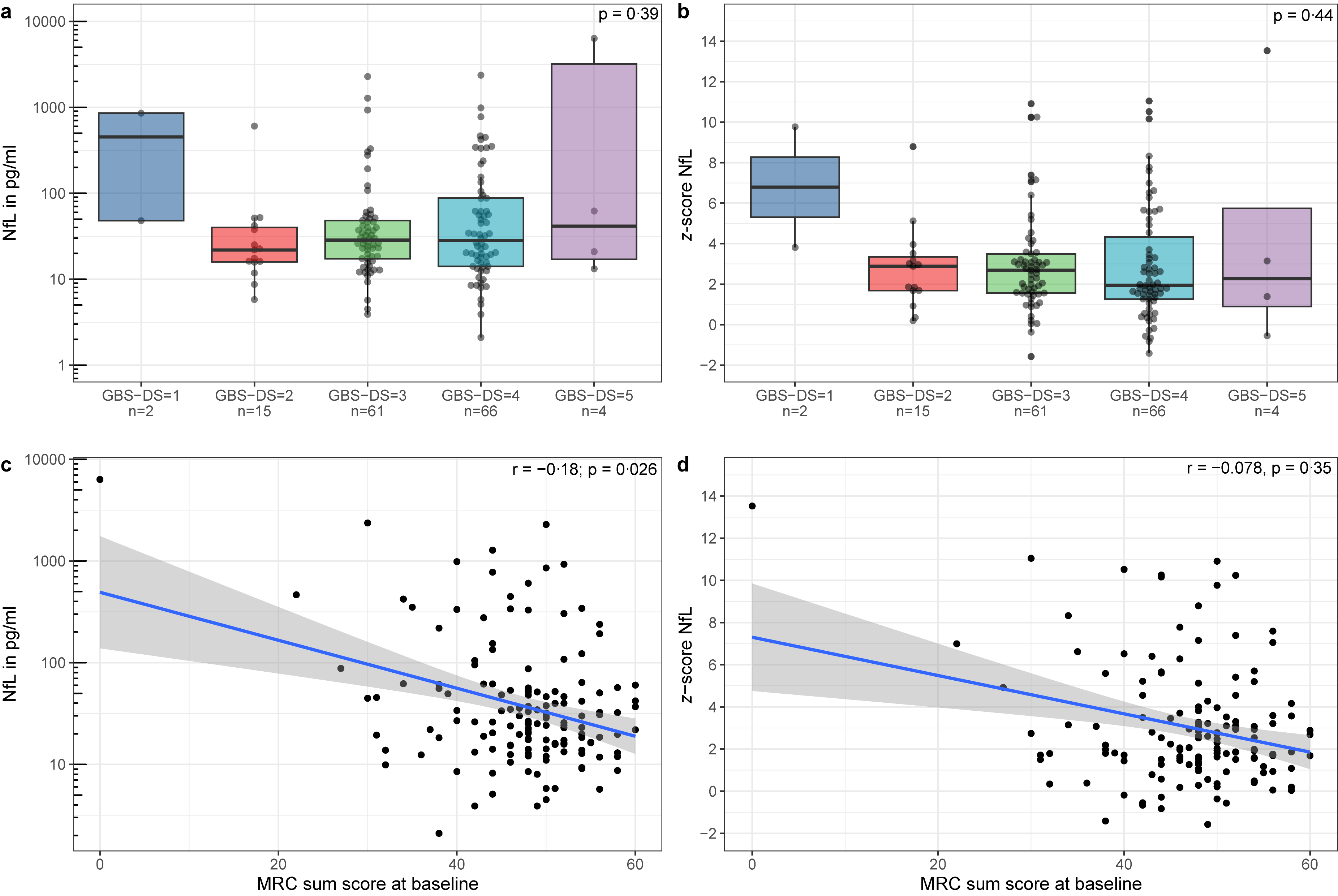


***Supplementary Figure S7*: Evolution of serum NfL over time in patients** **with different NCS variants**. The a) equivocal, b) axonal, and c) inexcitable NCS subtypes of GBS stratified by disease severity. The y-axis is on a log10 scale. For the inexcitable subgroup, GBS-DS of 1 and 3 did not occur in our dataset and are therefore not included. *Abbreviations:* NfL: Neurofilament light chain; MRC: Medical Research Council; GBS-DS: Guillain-Barré syndrome disability score.


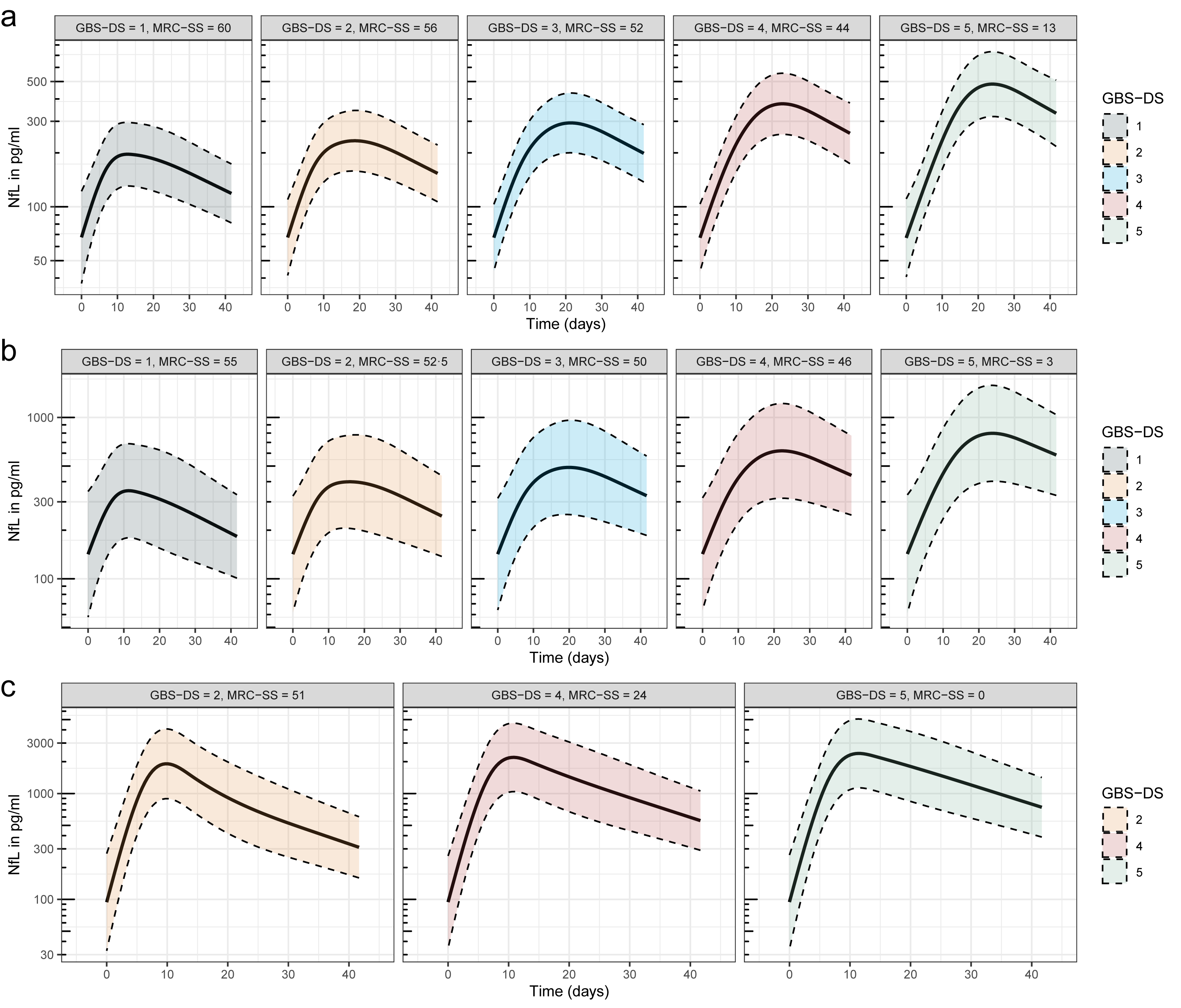


***Supplementary Figure S8***. **Time-to-event analysis based on age-corrected *z*-scores**. Stratification for *z*-score ≤ 2.5 or *z*-score > 2.5 at a) entry, b) week 1, c) week 2, and d) week 4. Outcome is the ability to walk unaided (GBS-DS < 3) for patients with GBS. Reported P-values were obtained using the log-rank test. *Abbreviations:* GBS-DS: Guillain-Barré syndrome disability score.


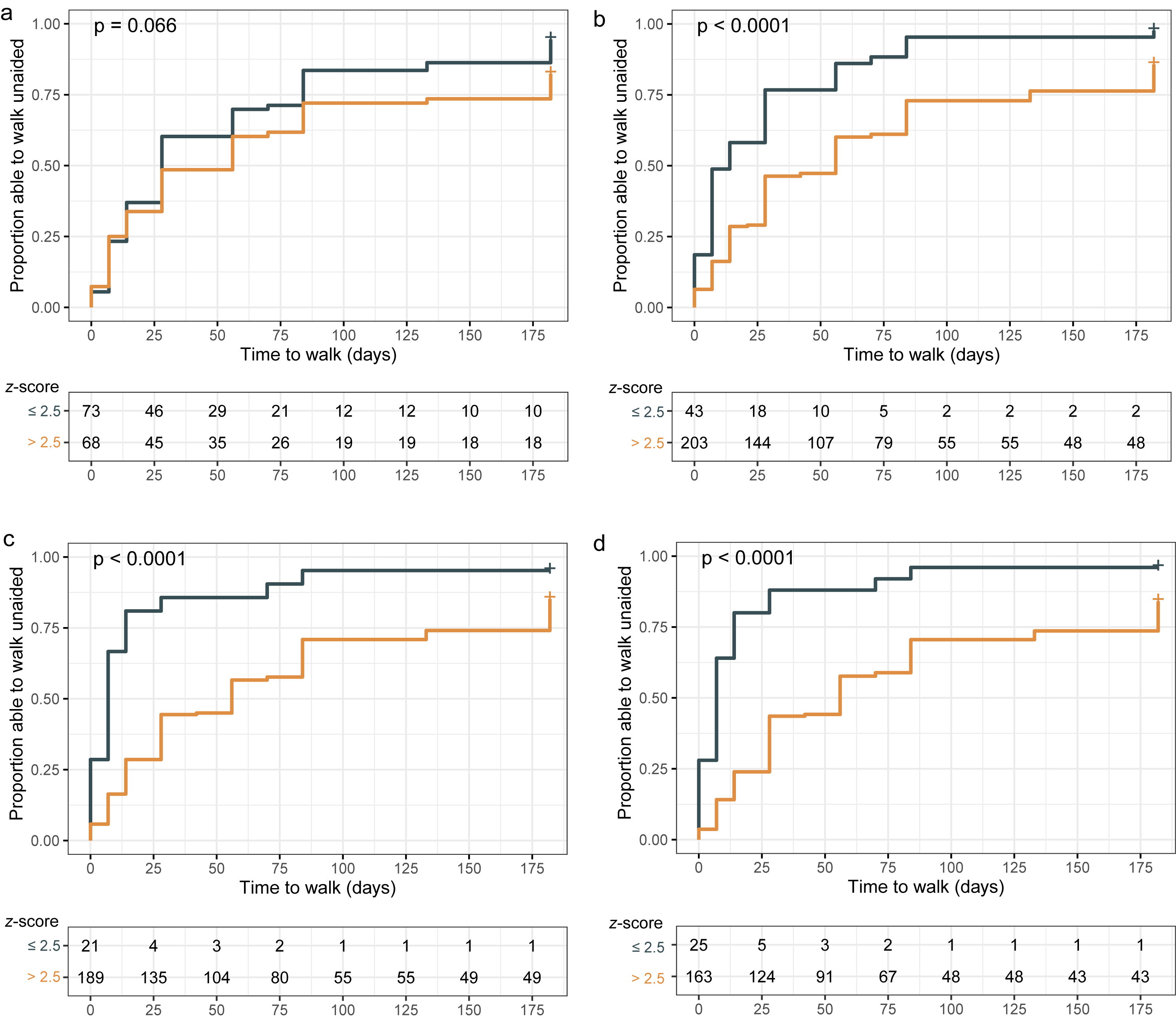


***Supplementary Figure S9***. **Association of adverse events with NfL levels**. Highlighted lines are patients that, a) are deceased b) experienced an embolic event, c) experienced pneumonia, d) or any serious adverse events during the course of the disease (in d; red lines = randomised patients, blue lines = non-randomised patients).


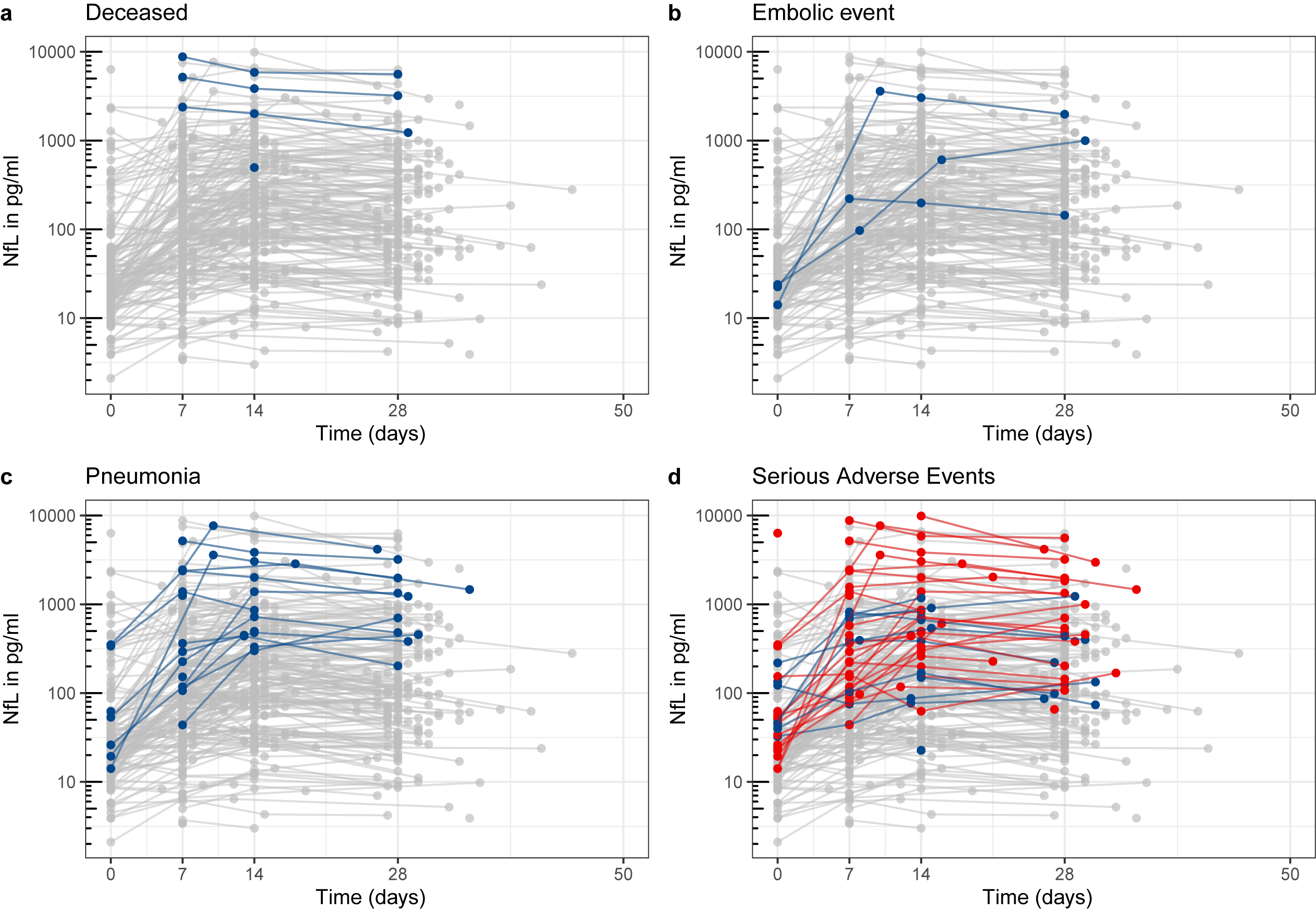


1. Vermunt L, Otte M, Verberk IMW, et al. Age- and disease-specific reference values for neurofilament light presented in an online interactive support interface. *Ann Clin Transl Neurol* 2022; **9**(11): 1832-7.

2. Benkert P, Meier S, Schaedelin S, et al. Serum neurofilament light chain for individual prognostication of disease activity in people with multiple sclerosis: a retrospective modelling and validation study. *Lancet Neurol* 2022; **21**(3): 246-57.

**References**
